# How does whole of government action address inequities in obesity? A case study from Australia

**DOI:** 10.1186/s12939-019-0913-6

**Published:** 2019-01-14

**Authors:** Melanie Pescud, Ginny Sargent, Paul Kelly, Sharon Friel

**Affiliations:** 1 The Australian Prevention Partnership Centre, Sax Institute, Ultimo, Australia; 2 School of Regulation and Global Governance (RegNet), College of Asia & the Pacific, Australian National University, Room 3.34, Coombs Extension Building, 8 Fellows Rd, Canberra, ACT, 0200 Australia; 3 Population Health Protection and Prevention, ACT Health, ACT Government, Canberra, Australia; 4 Australian National University Medical School, Canberra, Australia

**Keywords:** Health inequities, Obesity, Whole of government, Public policy

## Abstract

**Background:**

There are many factors across different sectors that contribute to inequities in obesity levels. This implies the need for action across different government departments and policy domains (hereafter referred to as whole of government multisectoral action). In this study we explored the public policy attention given to inequities in obesity using an Australian case study.

**Methods:**

Interviews were conducted with 33 stakeholders involved in the development and implementation of the whole of government Healthy Weight Initiative (HWI). A thematic analysis was undertaken to identify ways in which government policy makers and implementers explicitly or implicitly described how actions delivered through the HWI addressed inequities in obesity within the population.

**Results:**

The analysis revealed that the focus of the HWI was predominantly aimed at the general population, with minimal attention given to addressing the social distribution of obesity. The reasons for this were explained in terms of five themes: (1) rationale for a population wide approach; (2) when to apply an equity lens, (3) issues of government responsibility, (4) philosophically opposing concepts of equity, and (5) tensions across departments as a result of competing concepts of equity.

**Conclusions:**

It is important to create a shared understanding plus a concern for addressing inequities in public policy, regardless of whether or not a universal population-wide or a targeted approach is being applied. It is also important that policies and programs address the social distribution of obesity while understanding local contexts and needs. In striving to develop policy that brings an explicit focus on health equity, policymakers must consider the sociological, political, economic, and philosophical tensions at play between different policy actors and government departments, and identify how to navigate these without reverting to siloed working.

## Background

The considerable literature on the social determinants of health demonstrates that whole of government multisectoral actions, defined here as action across different governmental departments and policy domains (e.g., economic, social and health), generate and distribute income, goods, and services at the global, national, regional and community levels [1–4]. These policies affect the conditions of daily living and the practices that different social groups perform [5, 6]. Depending on the nature of these daily living conditions, different social groups are exposed differentially to health risk or protective factors, thereby resulting in health inequities [5, 6]. Health inequity, for the purposes of this study is defined as the presence of avoidable or remediable differences in health among people related to economic, social, demographic, or geographical differences [1–3]. In this study we explored the public policy attention given to addressing the social distribution of obesity using an Australian case study.

In many countries, people with less money, less education, insecure working conditions, and poor living conditions are more likely to eat unhealthy diets, not participate in sufficient physical activity, and have higher levels of obesity and the diseases associated with obesity [7–10]. Obesity is defined as having a body mass index of 30 or over [11]. Obesity is a complex policy problem. It is influenced by the food system and is also affected by economic, social, and cultural factors [5, 12]. These factors result in social stratification and influence the quality of conditions in which people live their lives and make choices [5, 12]. Within the food system, food production, manufacturing, retail, food services, and advertising determine what foods are available, where, and for what price. These in turn influence people’s knowledge, preferences, purchasing, cooking, and consumption practices [13–15]. Within the built environment, urban design, land use, modes of transport, public facilities, and market access affect people’s physical and sedentary behaviours [5]. Also of relevance to diet and physical activity are the nature of the physical and social experiences in early life; access to and quality of education, particularly that of females; the financial, psychosocial and physical conditions of working life, and the degree of social protection provided [8].

The multifactorial nature of the drivers of inequities in obesity levels implies the need for a whole of government policy response as well as actions by non-government organisations and industry. A focus on inequities in obesity also requires the responses to pay attention to the distribution of obesity across population groups and not just the average levels within a population [16–18]. Policies in government departments other than Health are needed to address the underlying social determinants of inequities in obesity, with the Health department playing an important stewardship role in enabling the necessary inter-departmental collaboration [19]. While whole of government policies to address obesity prevention are becoming increasingly commonplace [20], there appears to be little consideration of the distributional effects of these policies and also little action in policy areas outside health that may be responsible for policies that drive socioeconomic inequities.

### Policy case study

The policy case is the Healthy Weight Initiative (HWI). The HWI was launched in 2013 with the aim to halt the rising rates of overweight and obesity in the Australian Capital Territory (ACT) population [21]. The HWI used a whole of government approach to delivering policies and programs that aimed to improve food environments and active living environments. The HWI has been led by a central government agency, with technical input from health experts and a steering committee comprising members from seven government departments. Further details explaining the development and implementation of the HWI are published elsewhere [22]. The ACT is a city-state in Australia with an estimated population of 410,300 residents in 2016 [23]. Compared to other Australians, residents of the ACT have a higher life expectancy, earn more, are more educated, and have higher rates of employment [24]. Despite these socio-economic advantages, 63% of adults in the ACT are overweight or obese [24]. However, these averages mask the social inequities within the ACT, for instance, the level of obesity in the lowest socioeconomic group is 2.3 times higher than that experienced by those in the highest socioeconomic group [25]. The HWI was developed to address obesity across the whole population, thus providing an opportunity to study the ways in which the social distribution of obesity was addressed.

The aim of this study was, therefore, to provide empirical evidence on the ways in which policy attention to the social determinants of inequities in obesity manifests across a whole of government obesity prevention initiative (the HWI). Focusing on an Australian jurisdiction, the study identified ways in which policy makers and other key stakeholders explicitly or implicitly address the social distribution of obesity.

## Methods

A qualitative cross-sectional study design was adopted, with data collected using interviews with key stakeholders. These qualitative interviews were conducted with the dual purpose of collecting insights from stakeholders involved in the HWI to (1) inform an overarching evaluation, and (2) explore the policy attention given to addressing the distribution of obesity across the population. It is the latter that is the focus of this paper.

### Sample and recruitment

The government HWI co-ordinating unit compiled a comprehensive list of 33 key stakeholders who were involved in the decision making processes during the development and implementation of the HWI. Each stakeholder received a letter of invitation to an interview from the HWI co-ordinating unit. Two government staff declined but provided alternative contacts who were subsequently invited. The HWI co-ordinating unit provided the resulting list of stakeholders willing to be interviewed to the lead researcher, who subsequently made contact to book the interviews. Informed written consent was obtained before each interview commenced. Of the list of 33 potential interviewees provided, 28 agreed to participate (1 agreed to participate but experienced an illness and therefore had to withdraw) and an additional 6 were recruited through personal referral from interviewees. Subsequently 29 face-to-face interviews were conducted with 33 stakeholders (Table 1). Two were paired interviews and one was a group interview with three interviewees. The interviews were conducted between August and October 2016 and lasted between 45 to 60 min.

**Table 1:** Gender, sector and role of participating stakeholders (*n* = 33)

Category	Description	Quantity
Gender	Male	16
	Female	17
Sector/agency	Health department	6
	Central department	9
	Other departments	11
	Non-government agencies	7
Role	CEO	3
	Senior executive	6
	Manager	15
	Agency staff	9

Topics covered in the interview guide included: interviewees’ involvement with planning and implementing the HWI, ways in which interviewees thought their department could influence access to healthy foods or promote physical activity, and the presence and or absence of equity in policies and programs. Broader questions relating to the evaluation were also posed. In total, 27 of the interviews were digitally audio recorded and professionally transcribed, with handwritten notes taken in the remaining two interviews. As interviews were conducted among a small pool of stakeholders, to protect their anonymity participants are identified only by a code. Transcripts and coding were managed using NVivo11, a qualitative software analysis program.

### Data analysis

A thematic analysis [26], which is appropriate for use in applied research, was conducted to explore ways the participating policy makers and program implementers explicitly or implicitly described how actions delivered through the HWI addressed inequities in obesity within the population. A deductive coding schema was developed based on existing constructs and concepts from the literature including social determinants of health frameworks; equity/equality literature; and different policy approaches to equity (e.g., universal, proportionate universalism, targeting) [2, 8, 27]. Inductive topics which emerged from the interviews were added to the coding, such as economic rationalist arguments for policy-related decision making.

Coding and analysis were performed from the commencement of, and throughout, data collection. In order to conduct the thematic analysis, data were initially coded either sentence by sentence or paragraph by paragraph, depending on the variability of content [28]. As relationships or overlaps in the content of topics became clear, changes were made to the topics by either combining them, splitting them, or re-coding them to different constructs. Data saturation occurred prior to the end of coding (i.e. no new topics emerged and repetition of concepts was consistent), however all data were coded to ensure all participants’ views were included in subsequent analysis. Codes and full transcripts were read and re-read several times ensuring a high level of data immersion and enabling an in-depth interpretation of the data. Text and matrix searches were also used to explore differences and similarities in salient issues or perspectives according to the demographic characteristics of interviewees [29].

The lead researcher (MP) engaged SF in analytical conversations on a fortnightly basis in order to identify and then explore themes as they emerged. Both GS and PK were involved in the analysis and interpretation of data and refinement of themes at the completion of data collection. Refinement of themes also progressed as part of the writing and re-writing of the manuscript over time. As a result of the analysis, five themes emerged with pertinence to this study: (1) rationale for a population wide approach; (2) when to apply an equity lens, (3) issues of government responsibility, (4) philosophically opposing concepts of equity, and (5) tensions across departments as a result of competing concepts of equity.

An important aspect for establishing rigour in the findings was triangulation [30]. Four types of triangulation have been identified: (1) method triangulation, (2) theory triangulation, (3) data source triangulation, and (4) investigator triangulation [30]. The latter two were used in the present study whereby the views of various stakeholders were sought across all departments and by those at different levels within departments; and peer-review and input via continual discussion among the research team representing academia and government feeding into analysis and data interpretation.

## Results

The thematic analysis revealed that while the focus of the HWI was predominantly aimed at the average population level, some recognition was given to addressing the social distribution of obesity across the jurisdiction. Political, social, economic, and contested philosophical approaches to addressing obesity are apparent throughout the analysis. The results relating to each of the identified five themes are described in detail below:

### Theme 1: Rationale for a population wide approach

The official rationale for the population-wide focus of the HWI was articulated by two high level stakeholders who were influential in this decision. The main factors they identified were (1) they started with the evidence that overweight and obesity were highly prevalent (e.g., affect the majority of the adult population) and widely, not necessarily socioeconomically, distributed within the jurisdiction, (2) a concerted effort to prevent “othering” (labelling obesity a problem only associated with vulnerable or marginalised groups) the problem, (3) the understanding that population-based policies can be designed so that they have greater effect on more disadvantaged community members without the need to explicitly mention equity, and (4) the rationale that a focus on improving urban planning features would ensure that new infrastructure would be inclusive of all citizens regardless of socioeconomic status. Furthermore, other interviews revealed a confusion around the definition of what targeting meant across different departments, and this may have contributed to the population-wide approach.

While acknowledging the presence of socioeconomic impacts on obesity, one participant described the reasoning behind not articulating an equity focus in the aims of the HWI in terms of the high prevalence and broad distribution of obesity in the jurisdiction.

*I strongly argued for it not to be [an equity focus]. It wasn’t because I didn’t care about equity it was just I know this place. There’s no doubt there is a socioeconomic gradient for obesity as there is for most health risk behaviour and as there is for most health risk outcomes. So it wasn’t an intellectual rejection of that premise. But, we have two-thirds of the adult population overweight and we have a significantly above average income. Now those two figures cannot be consistent. (P27).*

During the development of the HWI, some stakeholders observed an “othering” of the problem by those in key policymaking positions and attributed this to the mainly middle class, bureaucratic perspectives being applied to understanding the development of overweight and obesity.

*I sit in meetings with fat bureaucrats on $200 grand a year who are continually talking about the poor people and how fat they are. It’s just another way of othering the problem. (P27).*

These perspectives contrasted with those of the two key decision makers in particular, who reiterated that high rates of overweight and obesity were not confined to lower socioeconomic groups. P27 went on to say:

*It’s not an others’ problem. This is a universal problem with the food supply. It’s poisonous basically and in chronic doses it’s poisonous right. For most people, me included. I have to say I’m overweight … Now if socioeconomic advantage means anything I really should be pretty thin. But it doesn’t outweigh the effects of the environment. (P27).*

These two interviewees expressed that labelling of overweight and obesity as a “*poor person’s problem*” was not only inappropriate, but by its nature, patronising and indeed inequitable. They therefore argued for designing population-based policies that had the potential for greater effect on more disadvantaged community members. By doing so, the policies could reduce inequities without the need to explicitly mention equity, and this was perceived to be a more palatable solution to addressing rising rates of overweight and obesity.

*I think what you have to do is construct a system which actually can have differential effects where the incentives are universal but they are not differentially applied. That is actually called the market generally speaking. So that’s why it [HWI] doesn’t contain an equity focus. (P27).*

The two interviewees also described the way in which actions to affect healthy eating and physical activity specific to disadvantaged groups were not necessary due to the urban planning features of the city, once again highlighting a context specific rationale. For example, it was noted that all residents have similar access to green space as it is abundant everywhere and that even those geographic locales where there are slightly higher pockets of disadvantage have good access to well-serviced shopping centres and supermarkets. This made drawing on learnings from other cities which incorporated equity into their policies difficult to translate as this jurisdiction was considered unique in nature.

*Look at the pockets of residents of X, very socially disadvantaged; actually no further from really good supermarkets than the very rich people who live next door. Access to green space? We’ve got nothing but green space. Most of the international experience with big cities doesn’t translate very well here. (P27).*

Many other interviewees also argued for a population-based approach whilst recognising the need to create policies and programs for community members experiencing the greatest need. However, these participants referred to achieving this through targeted approaches. These interviewees did not report the same sentiment as the two key decision makers referred to above; that is, they viewed overweight and obesity as a problem predominantly confined to lower socioeconomic groups within the jurisdiction. These interviewees suggested a range of approaches to addressing obesity through targeting disadvantaged community members.

*Normally where there’s a serious problem, governments don’t normally intervene at the population level, we intervene where the problem is most manifest … I would suggest you’d spend more on the disadvantaged and very overweight who may not have the private health insurance or the inclination to cover their own medical costs later in life. So I’d be more targeted at vulnerable/disadvantaged in the risk groups and I’d spend more time on the behavioural economics or the psychology of eating and good choices that people make. I’d get rid of soft drinks too. (P12).*

Justification for population-level approaches were also described in terms of increasing policy reach within a context of constrained human and financial resources.

*So we used to do that [target small community groups], but you’re talking about a group of 10 people. The money, the resources that you spend is huge for very, very limited impact. You do almost have to say, “Is that actually value for money and would we be better off working at a different level?” … That sounds really awful … We might be able to do things that influence work with those groups of people, but I don’t think we can put a lot of resources into working with very small groups of people. (P18).*

In this, and other interviews, there appeared to be moments of cognitive dissonance experienced by stakeholders. While many interviewees confidently upheld their views regarding the population-level focus of programs and policies based on the utilitarian argument to achieve the greatest impact for the greatest number, they still acknowledged that not addressing inequities was on some level, not intuitively right, as evidenced by the statement about the situation being “awful”.

The population level approach seemed to also be influenced by different and ambiguous understanding of the term ‘targeting’. Interviewees from the community services department viewed equity-focused work as that which targets small pockets within the community experiencing specific needs. Whereas those from the Health department viewed targeting as policy actions designed such that those experiencing the greatest disadvantage were benefitted most from the policy in terms of influence on health behaviours. Examples given were the cases of tobacco control pricing and soda taxation strategies [31, 32].

*There’s more and more evidence that we should be targeting disadvantaged individuals more and more and to support them, but the piecemeal* ad hoc *sort of stuff where some of the disadvantaged schools have got breakfast clubs and some of the centres have got supermarket tours or cooking classes or whatever else isn’t really enough. I think if you’re really going to make a difference in that space you need to make a systemic change. (P32).*

### Theme 2: When to apply an equity lens

The typical answer to the question “Are you able to tell me about any work that you are doing that has an equity focus?” was simply “*No*”, with interviewees explaining that equity did not feature in most project planning, reflecting responses given about the HWI as a whole, for example:

*We don’t target a particular group for a particular development … so it’s more overarching. (P19).*

In these discussions, some stakeholders added, unprompted, that they did however feel that equity should be something that receives further consideration. Equity was an inherent, albeit unintentional, feature of a small number of the discussed policies and programs. For example, policies such as those stipulating guidelines for healthy school canteen menus or healthy vending machines. Hospitals were noted as having substantial potential to influence more disadvantaged groups to a greater extent.

*I think when you’re working on the food environment a lot of it would be about equitable access simply because of what you’re trying to do. (P18).*

### Theme 3: Issues of government responsibility

As a way of unpacking their understanding of what an equity-focus might entail, interviewees were asked to consider what types of equity-focused policy and programmatic actions *could* be taken within their department, should the opportunity emerge to implement them. Only a handful of interviewees were able to provide suggestions to answer this question. This could reflect a lack of understanding of, or experience working on, equity-focused matters.

Within the urban planning domain, diversity of housing within higher socioeconomic areas was discussed as an avenue whereby disadvantaged members of the community could benefit, especially through greater access to employment. While employment and housing were not explicitly part of the HWI, some interviewees were cognisant of their influence on the distribution of obesity. For example, one interviewee spoke of the role of urban planning in reducing inequities through the ability of residents to live in a location that enabled them to walk or ride to work.

*… in terms of the broader equity question, what urban planning can do is make sure that we have mixtures of tenancies, like housing diversity. But in one area we have four jobs per resident, and we’re excluding more people moving in here … creating a disadvantage for people, about accessing work. So we’re making sure that there is housing diversity, that there is affordable housing, that there’s some public housing, that it’s not just a place for yuppies. (P03 and P04).*

The use of zoning restrictions for fast food outlets was highlighted as an important action for health equity as it would go some way to protect vulnerable populations from exposure to unhealthy food.

*… the fast food chains and all the poor food providers, do their socioeconomic analysis and put their restaurants on the journey home for poor people where they can easily access them. So you can go through and get crap food, and take it home, and then you’ve got dinner on the table. But can planning play a role in that? It could, but it’s going to be called social engineering. (P03 and P04).*

Throughout these discussions, it was apparent that issues of government responsibility were being highlighted, and there was some hesitation as to whether or not government had a role to intervene in some areas. For example, while a few stakeholders were able to describe potential equity-focused initiatives that their departments could take the lead on, others said they saw opportunities for equity-focused policy but stopped short of suggesting such actions take place as they did not believe it was the role of government to intervene in markets. One participant said:

*I think it needs to be led by the community and society that healthy eating and healthy lifestyles are what we want and then the private sector markets will respond and provide that. (P13).*

### Theme 4: Philosophically opposing concepts of equity

Differences amongst stakeholders in their understanding of equity became apparent when discussing the role of different departments and how equity could be built into policies or programs across all departments. Analyses revealed that the interviewees’ responses quickly focused on population-level actions to address nutrition, physical activity, and or weight, rather than the way in which these actions affect the distribution of obesity. When interviewees were asked to reflect on reasons why they automatically reverted to non-equity focused discussions, many reported that they simply do not think about it unless it is identified as a specific issue to be addressed and it is not “*front of mind*”. In many instances, the language and tone used to reflect upon the lack of equity focus conveyed a sense of guilt coupled with surprise that equity was not an explicit focus for the HWI.

*It sounds awful when I say it out loud, but I think we tend to think of inequity in terms of very traditional concepts around how staff should be treated, regardless of race, colour, religion,* etcetera*, but not in terms of inequity. (P05).*

It was also reported that many of the programs and policies implemented as part of the HWI would likely, by their nature, engage community members of higher socioeconomic status, rather than those experiencing the most disadvantage.

*So housing, transport, and urban planning are all relevant to my current role. And in housing and urban planning the two elements that catch me most on the food provision side are where people might want to grow a portion of food themselves and whether we’re actively thinking about that. But even where we do, we probably are pitching it more to higher socioeconomic class. So growing your own snow peas is something that people who have time and financially better off do rather than someone who does it to save money. (P07).*

Some stakeholders spoke in a very frank manner about the “*real classist element”* to conversations about equity by policy makers and the oftentimes “*patronising*” manner in which these unfolded.

*We have ascribed middle-class white values to some of these policy approaches and I do see that creeping in. (P08).*

*On average we’re incredibly healthy and wealthy … partly due to our unique approach to public housing where we have public housing throughout the suburbs, we have 12,000 public houses evenly spaced with some exceptions. There’s not a lot of the highly visible, in your face inequality that you might see in other cities, not based entirely on geography. You could take away a little bit of our extremely strong middle class welfare and try and spend some money on social inclusion initiatives, be it in the Health budget or other parts. Education is critical to that. Housing, welfare, social policy, urban planning … I think on those inequality is a problem but I think we need to spend more time acknowledging it and seeking it out because it’s not as visible as in other cities. (P12).*

These types of reflections provide insights into the way in which a lack of stark visibility and lack of acknowledgement of health inequities experienced by lower income groups, may have contributed to the absence of an equity focus in the HWI. Further, a few stakeholders felt that, in addition to their predominantly middle-class perspectives, there was a lack of understanding by some policymakers as to the structural factors influencing community members’ abilities to enact health promoting behaviour that could reduce obesity rates. This simplistic view of the factors leading to the emergence of inequities in obesity rates played out in the creation of policy that was not designed with equity in mind.

*The problem here is not that people misunderstand that what they’re eating is healthy, it’s the opportunity to eat healthy that is fundamentally different. They know it’s not good for them, they just don’t have a choice. I think that doesn’t necessarily play out very well in policy as I see it come through. (P08).*

An exception regarding an explicit equity focus were the social inclusion projects led by the community services department. However, despite equity-centred work being an explicit goal within this department, little was known about their work among many interviewees from other departments.

### Theme 5: Tensions across departments as a result of competing concepts of equity

The two dominant concepts of equity in programs and policies that became apparent through analysis of these interviews might be summarised in the following way: 1) that equity is achieved by reaching a high proportion of the population, and 2) that equity is achieved by targeted programs to address the distribution of obesity among vulnerable populations.

Although the HWI was described in terms of a population-level focus (the former conception), the Social Inclusion portion of the HWI, led by the community services department, was described in terms of addressing inequity (the latter conception):

*By targeting better nutrition and physical health as part of its broader social equity agenda, the government will build on the wide range of programs already in place to assist those experiencing disadvantage. (p. 15)* [21]*.*

The tension between these two conceptions of equity can be further understood using the frequently cited example of free swimming lessons that were offered to a small group of women. While the Community Service department believed in and were indeed proud of their equity-focused work relating to the swimming program, interviewees from other departments were not convinced of its merit citing a lack of value for money in terms of reach and effectiveness coupled with a philosophical opposition to downstream approaches to addressing overweight and obesity.

*I would say that’s something that hasn’t worked because they keep running swimming classes for 10 women and it’s great that they’re learning how to swim but there’s 10 of them! It’s not an obesity program. (P27).*

The tensions between these competing conceptions of equity became further apparent when interviewees expanded further on their differing expectations regarding program outcomes and whether success should be described for the individual or at the population-level.

*We’ve taught 100 women how to swim. Once. That’s a really wonderful thing. But is that going to change the population level of obesity? Absolutely not …*. *For those individual women … .it probably was a great thing. They had socialisation, they had physical fitness. But is it going to influence their individual health? Probably not. Is it going to influence the entire group of the community here? Probably not. Is it going to make any iota of difference to our zero growth in obesity target which is the target? It definitely isn’t. (P29).*

## Discussion and implications

Given the importance of equity as a public health policy goal [1, 2, 4, 33], the exploration of this aspect in relation to obesity and within a major whole of government initiative (the HWI) provides an opportunity for timely insights into how such a policy goal can be pursued when located alongside a number of other policy goals. The HWI was established under the premise of achieving the greatest good for the greatest number of people through its largely population-wide approach [21, 22]. In the study of the HWI, the factors affecting the absence or inclusion of equity in policies and programs were discussed and often exemplified by the presence of cross-departmental contentions regarding the use of population-wide approaches to public health versus targeted efforts to address inequities experienced by vulnerable groups within the community. While there are some generic and generalizable factors that can be applied to obesity prevention across settings [34], there are nuanced differences and details that come into play in different local contexts and in also addressing inequities [35].

This study revealed that although equity was not stated as an explicit focus of the HWI (except in the case of the work conducted by the community services department), all interviewees were aware of some level of obligation to address it. In many ways it could be suggested that while the end goal of reducing overweight and obesity in the jurisdiction was the same for all stakeholders, it was the means by which this was hoped to be achieved that were different. It is apparent from the current study that finding a way to capitalise on the common goal of obesity reduction is essential to creating effective policy and programmatic actions across the numerous departments involved in the HWI.

As demonstrated in the present study, progress can be hampered if equity is conceived in different ways by stakeholders. Therefore, the current study shows how a shared understanding of what is meant by equity is key to ensuring a policy goal is progressed. Indeed, unless equity is explicitly defined and prioritised as an issue to be considered, it is unlikely to factor into policy development and implementation.

It was apparent in this study that while there was intuitive appeal in addressing inequities, there was a predominant utilitarian way of thinking coupled with middle-class viewpoints which presented difficulties in negotiating the space. The jurisdiction being studied presents a curious paradox in that, in general, it experiences several advantages compared to other jurisdictions in terms of the social determinants of health, but this does not necessarily translate to better health outcomes [21]. Furthermore, while obesity is a population-wide issue in the jurisdiction, rates are higher in the lowest socioeconomic areas, which is consistent with national and international trends [16, 36, 37], and would suggest a need for equity-focused activities in order to redress such inequities.

Equity-focused actions, do however, need to be carefully considered, in order to avoid ‘lifestyle drift’ and ‘othering’ of the causes of obesity [38, 39]. ‘Lifestyle drift’ is a term used to describe policy actions that initially seek to tackle inequities via upstream approaches to addressing the social determinants of health, but then drift downstream to focus on factors at the individual level [38, 39]. Recent research suggests that government stakeholders become more cognisant of the occurrence of ‘lifestyle drift’ within their policymaking and in doing so, prevent othering of particular issues that are experienced to a greater degree by marginalised or vulnerable groups [40]. Arguably, the HWI has avoided othering the problem of obesity by taking a predominantly population-level approach.

In developing and implementing policies and programs to address population health issues that are inequitably distributed, like obesity, this study highlights the importance of also ensuring the needs of those most at risk are addressed in conjunction with using a universal population-wide approach. Priority must be given to ensuring inequities are not increased and giving attention to opportunities to decrease disadvantage [16]. Any concealment of poverty due to city planning may exacerbate disadvantage by rendering it invisible. Ensuring an appropriate mix of universal and targeted policies and programs fit for context, with sufficient resourcing, is key for effectively and respectfully addressing the distribution of obesity within populations [16].

As this analysis demonstrates, the political (e.g., policy contexts), social (e.g., class) and economic (e.g., finance and resources) tensions as well as the contested philosophical (e.g., inter-departmental tensions) approaches to addressing obesity creates challenges for cross-government approaches to problems that appear resistant to resolution [41–43]. These, coupled with the local contextual issues, makes addressing the distribution of obesity a complex challenge with no one-size-fits-all approach [5].

Systems science methodologies have shown promise as effective means for addressing obesity in ways that are fit for purpose within communities. For example, Allender and colleagues held systems mapping exercises with community members in order to identify solutions to obesity [35] and Friel and colleagues have created a systems diagram of the determinants of inequities in healthy eating [19]. Following a similar process could provide fruitful insights and learnings for whole of government strategies to address the social distribution of obesity within populations. Benefits of such methods include the ability for stakeholders coming together with different viewpoints to understand the reasoning behind seemingly ‘wrong’ ways of doing things in order that they may come to new points of compromise whereby key priorities can come into alignment [19, 35].

Our findings should be considered in light of the following methodological limitation. Our sample of interviewees were those who were involved in the HWI in some way, therefore there were no perspectives provided from those viewing the HWI from an external position. While we succeeded in our aim of interviewing a broad range of stakeholders from different departments, both inside and outside government and at different levels of the various agencies involved in the HWI, there may have been more contrasting views that were not represented in our sample.

## Conclusion

The present study has highlighted the need for policies and programs for addressing the distribution of obesity that are fit for local contexts. A general approach to applying an equity lens at the policy development stage could be to always ask ‘who benefits and who loses from each policy and program?’ Policymakers must consider the tensions at play between different value systems, understandings of equity, and institutional priorities, and identify how best to navigate these without reverting to siloed working. How equity is conceptualised must also be clearly defined and communicated from the outset, in the early stages of planning; this is also crucial for enabling effective evaluation efforts to better understand the value and differential impact of various interventions across socioeconomic strata. Finally, discussing the appropriate use of universal versus targeted approaches will be crucial to equitably addressing obesity in a whole of government context.

## Abbreviations

ACT: Australian capital territory
HWI: Healthy weight initiative
